# Effect of the Information Support Robot on the Daily Activity of Older People Living Alone in Actual Living Environment

**DOI:** 10.3390/ijerph18052498

**Published:** 2021-03-03

**Authors:** Jumpei Mizuno, Daisuke Saito, Ken Sadohara, Misato Nihei, Shinichi Ohnaka, Jun Suzurikawa, Takenobu Inoue

**Affiliations:** 1 National Rehabilitation Center for Persons with Disabilities Research Institute, 4-1, Namiki, Tokorozawa, Saitama 3598555, Japan; suzurikawa-jun@rehab.go.jp (J.S.); inoue-takenobu@rehab.go.jp (T.I.); 2 Faculty of Liberal Arts, Chuo Gakuin University, 451, Kujike, Abiko, Chiba 2701196, Japan; dsaito@fla.cgu.ac.jp; 3 National Institute of Advanced Industrial Science and Technology, 1-1-1, Umezono, Tsukuba, Ibaraki 3058560, Japan; ken.sadohara@aist.go.jp; 4 Graduate School of Frontier Sciences, The University of Tokyo, 5-1-5, Kashiwanoha, Kashiwa, Chiba 2778563, Japan; mnihei@edu.k.u-tokyo.ac.jp; 5 NEC Corporation, 1753, Shimonumabe, Nakahara, Kawasaki, Kanagawa 2118666, Japan; s-ohnaka@cp.jp.nec.com

**Keywords:** robot, older people, living alone, cognitive function, real life situations

## Abstract

Information support robots (ISRs) have the potential to assist older people living alone to have an independent life. However, the effects of ISRs on the daily activity, especially the sleep patterns, of older people have not been clarified; moreover, it is unclear whether the effects of ISRs depend on the levels of cognitive function. To investigate these effects, we introduced an ISR into the actual living environment and then quantified induced changes according to the levels of cognitive function. Older people who maintained their cognitive function demonstrated the following behavioral changes after using the ISR: faster wake-up times, reduced sleep duration, and increased amount of activity in the daytime (*p* < 0.05, *r* = 0.77; *p* < 0.05, *r* = 0.89, and *p* < 0.1, *r* = 0.70, respectively). The results suggest that the ISR is beneficial in supporting the independence of older people living alone since living alone is associated with disturbed sleep patterns and low physical activity. The impact of the ISR on daily activity was more remarkable in the subjects with high cognitive function than in those with low cognitive function. These findings suggest that cognitive function is useful information in the ISR adaptation process. The present study has more solid external validity than that of a controlled environment study since it was done in a personal residential space.

## 1. Introduction

The aging population in Japan was the highest in the world in 2015 and has remained higher than that in Europe and North America since then [1]. With the changing household composition, the ratio of older people living alone is gradually increasing [1]. Living alone, as opposed to living with family members or others, has been reported as a risk factor for difficulty in managing the instrumental and basic activities of daily living (IADL and ADL) [2], impaired cognitive function [3], depressed mood [4], low physical activity [5], social isolation [2,3], and disturbed sleep–wake rhythm [6].

Thus, it is expected that robots can support older people in maintaining their independence and compensating for a shortage of caregivers [7]. Considering the increasing number of older people living alone, it is possible to adopt robots instead of human resources to help older people to live independently [8]. Hence, an information support robot (ISR) system was developed with the capability of distributing the information required by older people through the Internet to assist them in maintaining their independence [9]. The information delivered at fixed times by the ISR facilitates the maintenance of daily routines for older people. Previous studies on the adaptation of information communication technology (ICT) to assist older people have proven it to be effective in curbing loneliness [10], wandering [11], anxiety, and depression [12]. Several robots have been developed and reported to influence older people’s cognitive function [13,14], physiological factors [15,16], behavioral and psychological symptoms of dementia (BPSD) [17], and relationships with their surroundings [15].

A previous study has pointed out that the required function of robots that support the independence of older people living alone is to help them to maintain a stable lifestyle [18]. Lifestyle stability and regulated routines are closely related to the sleep–wake rhythm, and there is evidence supporting the idea that an organized and stable lifestyle has a positive effect on sleep. For example, studies have shown that people with regular lifestyles have fewer sleep problems [19] and sleep more efficiently [20]. Zisberg et al. [21] suggested that increased stability in daily activities anticipated higher sleep quality in older people. A constant schedule of daily activity increased the amount of slow-wave sleep, which decreased with aging, in older people in assisting living facilities [22]. Thus, the relationship between lifestyle stability and good sleep practices has been clarified. Given that more than half of the community-dwelling older people have sleep disorders [23], there is a need for support focused on the regularity and stability of daily routines to promote the independence of older people living alone.

Although it has been clarified that robot assistants have had an impact on older people, the literature concerning the effects of robots on the regularity and stability of daily routines is limited. It has been suggested that robots contribute to the stability of daily activities [9,13]; however, there is still no conclusive evidence. In addition, the limited adoption of robots in actual settings can be attributed to the lack of demonstrated effects [24,25]. Most studies were conducted in hospitals, nursing homes, care facilities, and living lab settings [14,15,16,17,26,27,28]. Abdi et al. [29] pointed out that future studies on robot assistants should be more conscious of real-life scenarios. One study was conducted in residential spaces but the results could not be generalized due to the limited sample [30]. Two other studies demonstrated the effects of robots on quality of life and medication adherence in real-life situations [31,32]. However, these studies have not objectively represented the situation of older people living alone in all details since the outcome measures were dependent on self-reporting.

Due to this, it remains unclear if the robot facilitates stable daily activity for older people living alone, especially if the robot encourages older people living alone to alter their sleep–wake rhythm. Furthermore, it is unclear whether the robot has an influence on the sleep–wake rhythm of older people living alone homogeneously, regardless of the cognitive function level.

The purpose of this study was to confirm the effects of the ISR on daily activities, especially regarding sleep, of older people living alone. We simultaneously investigated whether the differences in the effects depended on cognitive function by measuring the cognitive function of all the subjects. It was hypothesized that the ISR would facilitate stable daily activities for older people living alone and alter sleep patterns since lifestyle regularity is associated with morning tendencies [33]. It was also hypothesized that the effects would be dependent on cognitive function since there could be gaps between the cognitive function of older people and the function of the prescribed robots [34]. If the effects of the ISR in altering the daily activity of older people living alone depend on cognitive function, it would show that considering the users’ cognitive function levels is useful when introducing a robot.

### Related Work

Several studies on robots for people with dementia and cognitive impairment have suggested that robots allow them to become more independent. A PARO is a famous animal robot for caring for older people, and it has been shown to achieve positive effects on stress [15], blood pressure [16], and BPSD [17]. Nonetheless, PARO cannot distribute the information required to assist older people in their daily routines.

Focus group discussion has identified voice operations as a requirement for human–robot interaction [7]. Home-based healthcare robots with voice-operating systems have been developed. A Sillbot robot was developed to assist older people in their daily activities, including medication reminders and informed daily schedules [35]. Kompai [36] and Ryan [37] are companion robots with verbal and touchscreen interactions for older people, while it remains unclear whether these robots have an impact on the daily activity of older people due to the robots being in the developmental phase or lacking verification. Broadbent et al. [31,32] have conducted experiments involving older people and robots in real-life settings and found that the robots were likely to reduce depression and improve medication adherence and quality of life. Other studies that investigated the relationship between robot assistance and sleep pattern have demonstrated that the intervention of Kabochan, a human-type communication robot, increased sleep duration [13], whereas PARO intervention did not affect sleep duration [38]. Consequently, there is no consensus on the effects of robots on the daily activities of older people.

## 2. Materials and Methods

### 2.1. Subjects

The study subjects were 14 older people who had been living alone. Mean age of all the subjects was 82.8 ± 4.9 years. Eight and six subjects lived in paid facilities based on self-support and in their own houses, respectively. All subjects were women. They had been verified by the Japanese long-term care insurance, which is a system to facilitate older people who need long-term care to lead an independent daily life at home as much as possible. The system is such that people over the age of 40 become insured, pay insurance premiums, and can use the service when long-term care is required. In order to receive the service, people need to apply to the municipality and be certified as being in need of long-term care or support. All the subjects had been given a type of formal care. Four of them had been diagnosed with dementia, three with Alzheimer’s, and one with vascular dementia. The other subjects had not been diagnosed with a specific dementia. We included subjects with histories related to memory or orientation disorders. The informal or formal caregivers of the subjects provided information about the memory or orientation disorders of the subjects. We excluded those who had hearing impairments and would have had difficulty understanding the information provided by the ISR. Informed consent was obtained from all subjects and their families. The study was approved by the ethics committee of the National Rehabilitation Center for Persons with Disabilities.

### 2.2. Procedure

**Figure 1:**
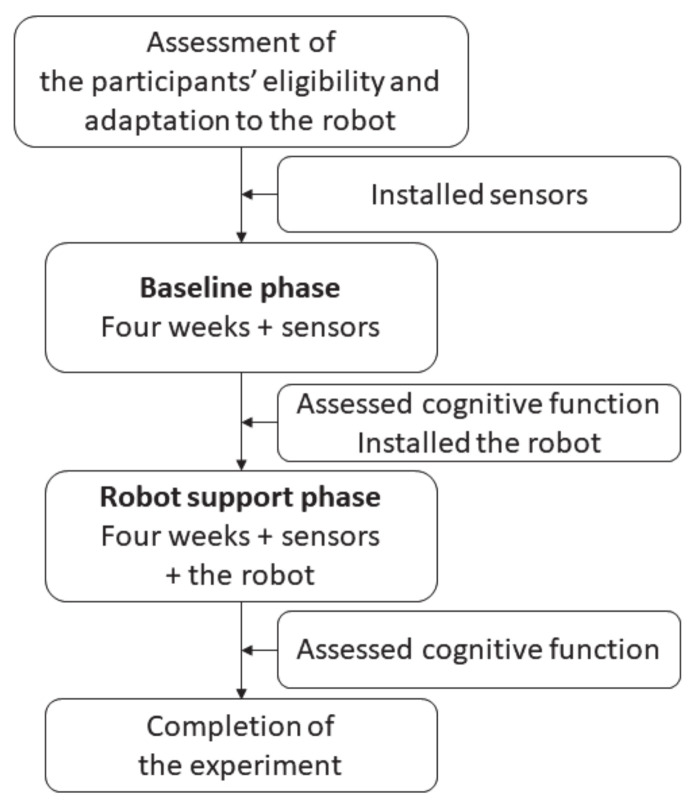
Procedure overview.

We applied a pre–post comparison study consisting of two phases: a baseline phase (BLp) and a robot support phase (RSp). The comparison was performed to verify the effects of the ISR on the daily activities of subjects living alone. After collecting the activity data in the BLp for four weeks, we implemented the ISR system and collected activity data in the RSp for another four weeks. All subjects interacted with the ISR before starting the experiment to confirm their eligibility and adaptation to the ISR. We assessed the subjects’ cognitive function before and after the RSp. The study procedure is illustrated in Figure 1.

### 2.3. Information Support Robot

**Figure 2:**
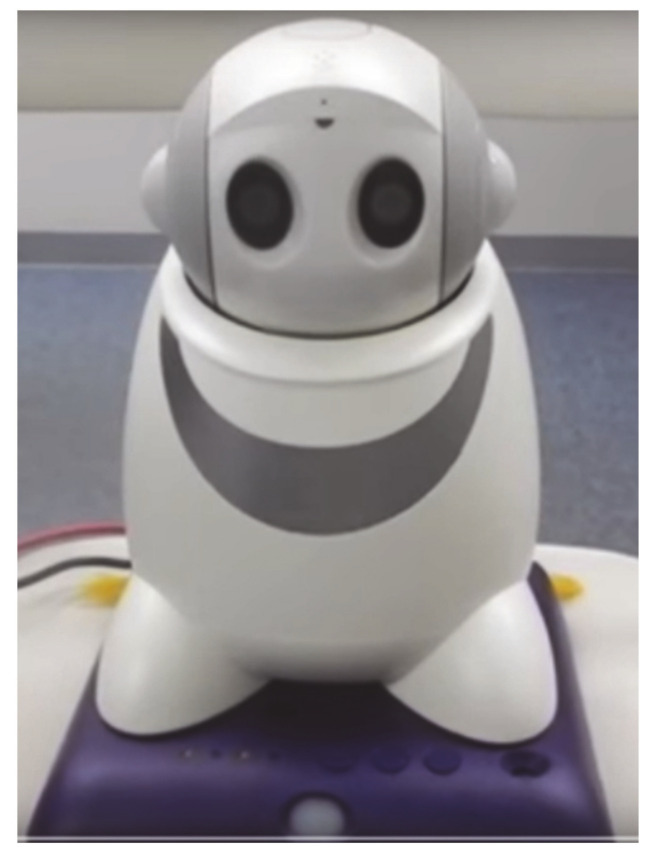
PaPeRo i robot, produced by the NEC Corporation.

We used PaPeRo i (NEC Corporation, Tokyo, Japan) as the platform for the ISR system (Figure 2). The robot was 288 mm tall, 255 mm wide, and 255 mm deep. It was equipped with a microphone, camera, head motion, and a light indication function; only head motion and light indication were used in the present study to interact with the subjects.

**Figure 3:**
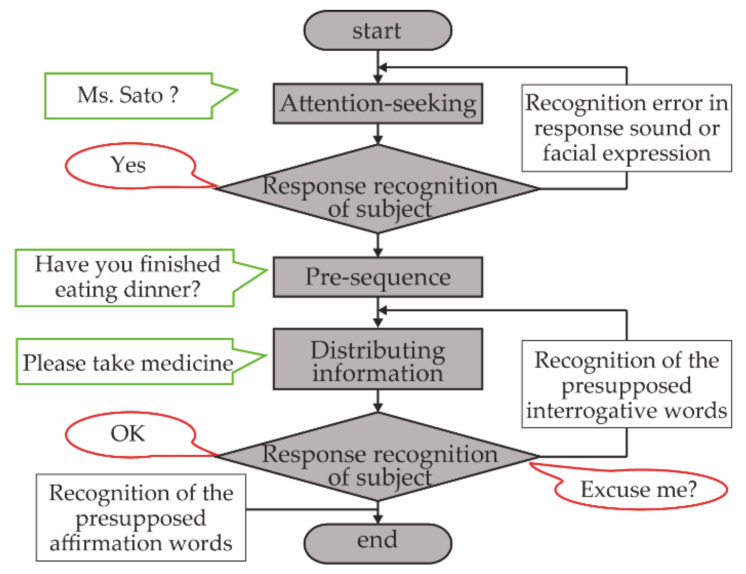
Information support algorithm that was developed to reliably convey information to older people.

This ISR system employs an information support algorithm. First, the robot calls the user’s name. Then, if there is no response, the robot calls the name again. Next, the robot distributes the information that is most desired to be communicated after the preceding information. At this time, if interrogative words or an error is recognized, the information will be distributed again. If affirmative words are received, the information support will be terminated with the closing word (Figure 3) [9]. A talking pattern allowed the older people to listen easily according to the characteristics of their cognitive impairment [39]. It was clear that the algorithm and talking pattern certainly allowed older people to obtain information. We determined the information to be spoken by the robot before hearing from the subjects, their families, and their formal caregivers. The information was then inputted to the robot through the Internet so that it could speak at specified times to support the subjects. The input information included content that would help the subjects to live independently. For instance, the information could remind them of the time to wake up, go to bed, eat a meal, take medicine, go out, watch a TV show, and take out garbage.

### 2.4. Measures

**Figure 4:**
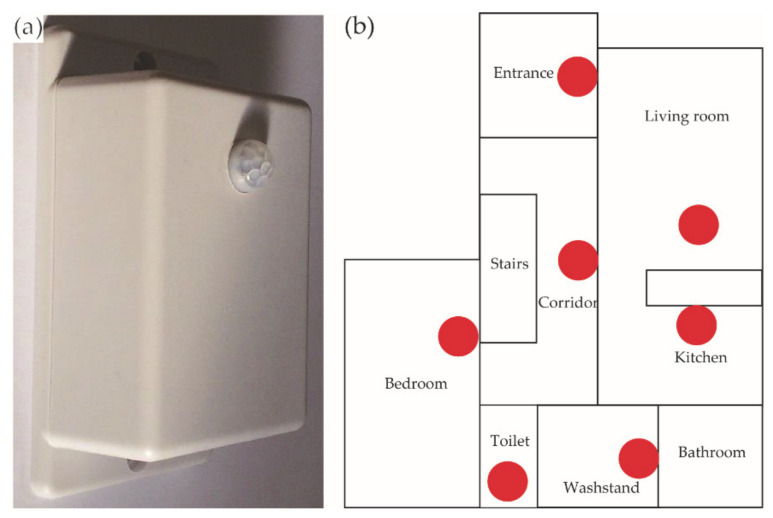
Infrared sensor and installation drawing. (**a**) Infrared sensors (HIRO ICT Inc.; L90.0 × W50.0 × D30.0 mm) were used to evaluate the daily activity of older people. (**b**) Example of the sensor installation. Red dots represent the points where the sensors were installed.

The subjects’ daily activities were recorded using infrared sensors installed in their indoor environments. The sensor (HIRO ICT Inc., Kanagawa, Japan) was 50.4 mm × 70.0 mm × 38.0 mm (Figure 4a), and it was designed to react to heat sources. On detecting a heat source, the sensor switches on. Subsequently, when the heat is not detected for 10 s, the sensor switches back off. A Bluetooth Low Energy device was used to connect the sensor to a tablet, which was the control device for the sensors. For each subject, 5–8 sensors were used to assess activity in their rooms, and the sensors were attached to a wall or ceiling in the living room, bedroom, kitchen, lavatory, entrance, toilet, and corridor, etc. Figure 4b illustrates an example of a floor plan with the installed sensors. As infrared sensors were used, the subject’s needs for privacy protection were met. Moreover, the sensor was easy to install and maintain, making it suitable for long-term experiments in home environments.

We calculated the following indices using the data obtained from the sensors: (i) wake-up time, (ii) bedtime, (iii) duration of sleep, (iv) frequency of all the sensors’ firings in the daytime, and (v) frequency of all the sensors firings at night. Table 1 lists the definitions of these indices. Indices (iv) and (v) were calculated based on the raw sensor data. The following other daily activities were also calculated: (vi) frequency of short outings, (vii) frequency of long outings, (viii) frequency of toilet use in the daytime, and (ix) frequency of toilet use at night. Table S1 lists the definitions of (vi)–(ix).

In addition, we assessed each subject’s cognitive function using the Mini-Mental State Examination-Japanese (MMSE-J). The MMSE-J test was translated from the MMSE by Dr. Sugishita and has been investigated for reliability, predictive validity, and specificity [40]. The test results were calculated from 0 to 30 points. The cutoff value between healthy and cognitively impaired groups was defined as between 23 and 24 points. The predictive validity of the classification between 23 and 24 points has been well studied. We classified the subjects by cutoff values during the statistical processing. Subcomponents of the subjects’ cognitive function were assessed using the Japanese version of the Neurobehavioral Cognitive Status Examination (COGNISTAT), which has been studied for reliability and validity [41,42]. This included 10 subcomponents of cognitive function: orientation, attention, comprehension, repetition, naming, constructional ability, memory, calculation, similarities, and judgment. First, an examiner asked the question in each subcomponent and assigned it scores, which were raw scores. Next, the examiner used a conversion table in the manual to calculate the standard scores from the raw scores. Finally, the cognitive function of each subcomponent was represented as a standard score in the range of 0 to 12 points. COGNISTAT adopts a standardization step to allow comparisons between subcomponents. Eight points were located at the boundary between the normal and impaired areas. A score above 8 points means healthy, a score of 8 points or less means disability; the lower the score, the more severe the disability. The COGNISTAT allowed us to visually identify the abilities that were retained and those that were impaired.

### 2.5. Statistical Analyses

We assessed cognitive function using the MMSE-J and COGNISTAT immediately before and after the RSp. Ages between the ≤23 and ≥24 groups were compared using an unpaired *t*-test; the COGNISTAT subcomponent scores between the ≤23 and ≥24 groups in each BLp and RSp were compared using the Mann–Whitney *U* test. In addition, we used the Wilcoxon test to compare the COGNISTAT subcomponent scores between the BLp and RSp in the ≤23 and ≥24 groups. As for the indices of daily activity, after averaging the indices’ data for each pre–post period, we subsequently performed a paired nonparametric analysis to compare the differences between BLp and RSp. The significance level for each statistical analysis was 5%. All the analyses were performed using IBM SPSS statistics 25 software (IBM Corporation, Armonk, NY, USA).

## 3. Results

### 3.1. Subjects’ Cognitive Characteristics

Table 2 presents the mean age and cognitive function data of each subject group. We obtained valid data for 14 subjects who used the ISR system until the end of the experiment. The mean age of all the subjects was 82.8 ± 4.9 years. The subjects could be divided into two groups: ≤23 and ≥24 points by MMSE-J. Each of the ≤23 and ≥24 groups was composed of seven persons. The mean ages of the subjects in the ≤23 and ≥24 groups were 82.1 ± 6.1 years and 83.4 ± 4.2 years, respectively. The oldest age of the subjects in the ≤23 group was 93 years old and the youngest was 76 years old. The age ranges of the subjects in the ≥24 group were from 88 to 76 years old. There was no significant difference between the two groups with respect to age (*t*[1, 2] = −0.46, *p* = 0.04).

There were significant differences between the groups in terms of the COGNISTAT subcomponent scores. The orientation in BLp and RSp (*Z* = −2.54, *p* = 0.011; *Z* = −2.98, *p* = 0.002, respectively) was significantly different between the groups. Comprehension and naming (*Z* = −2.87, *p* = 0.008; *Z* = −2.20, *p* = 0.035, respectively) in RSp were significantly different between the groups. The construction ability (BLp: *Z* = −3.04, *p* = 0.001; RSp: *Z* = −2.97, *p* = 0.002) was significantly different between the groups for both BLp and RSp. In addition, memory (BLp: *Z* = −2.55, *p* = 0.011; RSp: *Z* = −2.94, *p* = 0.002) was significantly different between the groups for both BLp and RSp. The similarities in BLp (*Z* = −2.22, *p* = 0.026) were significantly different between the groups. By making comparisons within the groups, a significant difference was found with respect to similarities for the ≤23 groups (*Z* = −2.02, *p* = 0.026).

### 3.2. Comparison of the Indices of Daily Activities

We compared the indices between the groups at the time of BLp in order to compare the states of both groups before introducing the ISR. As a result, no significant differences were found in the three indices: wake-up time, bedtime, and sleep duration in BLp.

According to the daily activity defined by the sensor data (Table 3), wake-up time was significantly earlier in RSp compared with BLp in the ≥24 group (*Z* = −2.03, *p* = 0.043), not in the ≤23 group. Moreover, sleep duration significantly decreased in the ≥24 group (*Z* = −2.37, *p* = 0.018). There was no significant difference in bedtime (*Z* = −0.85, *p* = 0.40). On the other hand, little change was seen in the wake-up time, bedtime, and sleep duration of the subjects in the ≤23 group (*Z* = −0.34, *p* = 0.74; *Z* = −0.51, *p* = 0.61; *Z* = −0.17, *p* = 0.87, respectively). As can be seen from other indices of daily activity, frequencies of short or long outings did not show significant differences; frequencies of toilet use during the daytime and at night also showed no significant differences between BLp and RSp for either group (Table S2).

**Figure 5:**
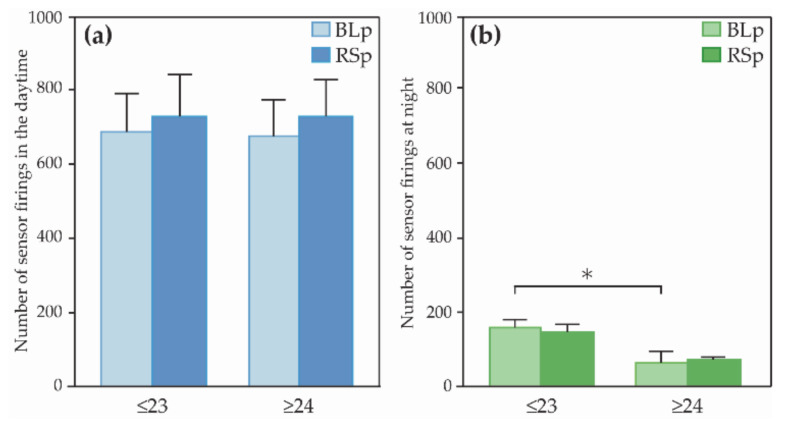
Comparison of (**a**) the number of sensor firings in the daytime and (**b**) the number of sensor firings at night. Error bars indicate standard error, and asterisk indicates that *p*-value was less than 5%; BLp: Baseline phase, RSp: Robot support phase

Figure 5 shows the analysis of the number of sensor firings. The left and right graphs present the daytime and nighttime, respectively. The number of sensor firings at BLp was analyzed using the Mann–Whitney *U* test in order to compare the state of both groups before introducing the ISR. The results showed that the number of sensor firings at night in the ≤23 group was significantly higher than that in the ≥24 group (*Z* = −2.36, *p* = 0.017, *r* = 0.63)

As can be seen from Figure 5, it showed that there was no statistically significant difference, although the number of firings during the daytime was increased in the ≥24 group (*Z* = 1.86, *p* = 0.063, *r* = 0.70) Additionally, the counterparts in the ≤23 group showed no significant differences between BLp and RSp (*Z* = 0.68, *p* = 0.50, *r* = 0.26). Subjects in both ≤23 and ≥24 groups showed no significant differences in the number of sensor firings at night (≤23: *Z* = 0.51, *p* = 0.61, *r* = 0.19; ≥24: *Z* = 0.17, *p* = 0.87, *r* = 0.06).

**Figure 6:**
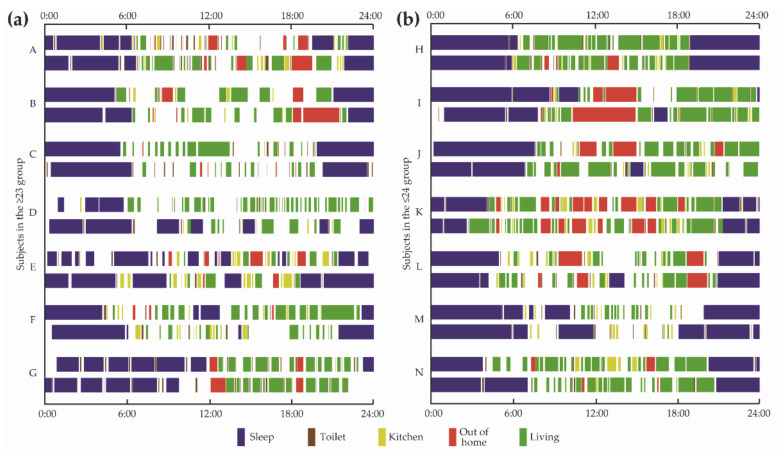
Daily activity maps of all the subjects (a) in the ≤23 groups and (b) in the ≥24 groups, visualized from sensor data. The upper bar of each subject is a typical activity map at the BLp. The bottom bar of each subject is a typical activity map at the RSp.

Figure 6 illustrates the daily activity maps of all subjects in the ≤23 and ≥24 groups. Daily activities were tagged and color-coded from the sensor data. Activity maps of some subjects in the ≤23 group show irregularity in the sleep–wake rhythm and a tendency to use the toilet at night for both BLp and RSp. On the other hand, activity maps of some subjects in the ≥24 group show regularity in the sleep–wake rhythm, and a tendency for earlier wake-up times during RSp, and a few subjects show an increasing frequency of going out during RSp. Blank areas in color bar indicate the activities that cannot be defined. For example, if multiple sensors are simultaneously fired for several minutes, no activity is defined.

## 4. Discussion

In the present study, we introduced an ISR into the actual living environment to determine the effects of the ISR on the daily activities of older people living alone based on cognitive function. Based on the analysis of the data obtained, the ISR altered the wake-up time and sleep duration of subjects with high cognitive function rather than those with low cognitive function.

Regarding the validity of dividing the subjects into two groups according to MMSE scores, we found that the orientation, construction ability, memory, and similarities in the COGNISTAT subcomponent score in BLp were significantly different between the ≤23 and ≥24 groups. A study [43] showed that MMSE scores were significantly correlated with orientation on the COGNISTAT subcomponent score. Matsuda et al. [42] reported that orientation, construction ability, memory, similarities, and judgment are useful to compare the healthy individuals with impairments. The characteristics of the groups divided by MMSE scores in the present study were consistent with those of previous studies.

The most notable findings of this study were an earlier wake-up time and reduced sleep duration in the ≥24 group. This suggests that the ISR has an impact on daily sleep patterns. D’Onofrio et al. [44] suggested that ICT systems facilitate some aspects of the activities of older people. However, there is little evidence that ICT and robot systems directly alter sleep state. The ISR used in the present study was unable to wake up the subjects but could provide information at specified times. Therefore, the ISR may have enhanced the regularity of daily activities and indirectly influenced daily wake-up times and sleep duration. These findings are consistent with a previous study showing that people with regular lifestyles tend to be morning types [33].

Corroborating the aforementioned findings, subjects in the ≥24 group demonstrated increased sensor firing during the day, which correlates with increased daytime physical activity in the house. Evidence shows that physical activity improves sleep quality [45]. Furthermore, several sociodemographic characteristics, such as age, gender, income, and education, are associated with physical activity [46]. Living alone at an old age reduces physical activity [47]. Additionally, a study has reported that reduced physical activity among older adults was due to a lack of motivation [48]. In our study, subjects in the ≥24 group showed increased physical activity after using the ISR compared with those in the BLp. Thus, information provided by the ISR allows older people to improve their daily routines and activity levels. In contrast, the daily activity of the ≤23 group showed no significant difference between BLp and RSp. This was consistent with a previous study that demonstrated that the robot, PARO, had no impact on the sleep patterns of people with dementia [38]. Additionally, it has been reported that the ADL patterns between healthy patients and those with dementia were significantly different [49]. This difference may have influenced the results of our study. Namely, the impact of the ISR on the daily activities of older people could vary depending on cognitive status. Since several previous studies tended to target general end-users with various degrees of cognitive function, a lack of specificity has led to limitations in the adoption of robots [25]. Therefore, it is important to consider cognitive functions in introducing an ISR suitable for older people with cognitive impairment.

The amount of sensor firing and the frequency of nighttime toilet use did not change between BLp and RSp. This supports the finding that sleep was not interrupted. An earlier study [50] suggested that sleep quality tends to decrease with age, and for people over 60, living alone exacerbates this problem. Sleep problems lead to a lower quality of life for older people living alone [51]. Therefore, the finding that ISR could indirectly affect sleep provides a useful perspective in promoting the independence of older people living alone.

Finally, the present study had more solid external validity as it placed the ISR in personal residential spaces rather than in controlled environments. Studies on robots for caring for older people should pay attention to a translation into real situations since it is difficult to determine the applicability of the robot without valid assessments that are closely related to the daily living [29]. Our study offers useful information about adapting an ISR to older people with cognitive impairment because the effects of the ISR were validated in an uncontrolled environment.

### Limitation

In the present study, the ISR altered the daily activities of community-dwelling older people. However, it should be noted that the results were interpreted with caution since the present study has addressed a small sample size. In addition, the effects of getting used to the robot are not reflected. Although this point is beneficial for robot studies, the present study did not reveal the dynamics of the observation along the time axis due to the small sample size. The results from this study included women only; therefore, it was unclear whether the ISR influences the daily activities of male subjects as well as women.

There are some limitations of the present study regarding the outcome measures. The MMSE-J scores were not corrected for age and education. Although we adopted the cutoff values between 23 and 24 points, people with the MMSE-J of ≥24 points also might have symptoms of mild cognitive impairment. Thus, further investigation is needed to determine the effect on cognitive levels. It was difficult to interpret whether the subjects’ sleep quality improved in the present study since the quality of sleep has not been adequately evaluated. Future study needs to be conducted on a larger sample size and measuring sleep quality to gain generalizable information on the effects of the ISR. The utmost attention should be paid to the fact that the present study was conducted in Japan, which is a country of advanced technology and with one of the largest ageing populations worldwide. Thus, these results should be interpreted against the background of Japan.

## 5. Conclusions

The present study demonstrated that an ISR influenced the daily activities of older people in their actual living environment. Older people who maintained their cognitive function, that is, those with MMSE-J scores of ≥24, showed faster wake-up times, reduced sleep duration, and increased amount of activity during the day after introducing the ISR. The results suggest that ISR is beneficial in supporting the daily independence of older people living alone. The effects of ISR were particularly dependent on cognitive function as the ISR changed the daily activities of older people who maintained their cognitive function. These results demonstrate that it is important to consider the cognitive function of older people when installing robots in their living environments. Moreover, future research involving in robots that facilitate older people’s independence should be conducted for a longer period in a real-life setting. The lifestyle of older people varies with their cognitive and physical functions, which change with time. Thus, the robot adaptation process should always be conscious of the real-life situation.

## Figures and Tables

**Table 1:** Definitions of the indices involving sleep and the indices of the amount of activity.

No.	Index	Definition
i	Wake-up time	Last sleep end time before 12:00
ii	Bedtime	First sleep start time after 18:00
iii	Sleep duration	Total time for each sleep from 18:00 on the day to 12:00 on the next day. In the case of the other sensors outside the bedroom fired between sleep periods, it is also considered as sleep if the sleep-to-sleep interval is less than 10 min.
iv	Number of sensor firings in the daytime	Number of sensor firings data not during sleep.
v	Number of sensor firings at night	Number of sensor firings data during sleep.

**Table 2:** Age and cognitive function data for two groups divided by the Mini-Mental State Examination-Japanese (MMSE-J) scores.

		≤23 Group (n = 7)		≥24 Group (n = 7)		Effect Size			
Age ^a^ (Range)		82.1 (76–93)		83.4 (76–88)		0.13 ^x^			
						Intra ^y^		Inter ^z^	
		Baseline Phase (BLp)	Robot Support Phase (RSp)	BLp	RSp	≤23	≤24	BLp	RSp
Mini-Mental State Examination-Japanese (MMSE-J) ^a^		19.9 (14–23)	19.3 (16–22)	27.7 (24–30)	28.6 (26–30)	0.10	0.43	−	−
The Japanese version of the Neurobehavioral Cognitive Status Examination (COGNISTAT) ^b^	Orientation	6.0 (1.5–8.5)	5.0 (0.5–8)	10.0 (9.5–10)	10.0 (10–10)	0.00	0.00	0.68 *	0.80 *
	Attention	6.0 (3–10)	8.0 (3.75–10)	8.0 (4.5–10)	3.0 (1–6.5)	0.38	0.56	0.04	0.40
	Comprehension	7.0 (5.5–10)	7.0 (4.75–7)	10.0 (10–10)	10.0 (10–10)	0.65	0.00	0.60	0.77 *
	Repetition	11.0 (8.5–11)	9.0 (7–11)	11.0 (8.5–11)	11.0 (8.5–11)	0.17	0.22	0.06	0.17
	Naming	9.0 (7–9)	7.0 (7–8.5)	9.0 (9–10)	10.0 (9–10)	0.00	0.38	0.42	0.59 *
	Constructional ability	7.0 (6–8)	7.0 (7–7.75)	11.0 (10–11)	11.0 (10–11)	0.21	0.00	0.81 *	0.79 *
	Memory	7.0 (6–7)	5.5 (5–6.75)	9.0 (8.5–10)	10.0 (8–10)	0.62	0.22	0.68 *	0.78 *
	Calculation	8.0 (5–10)	7.0 (4–10)	10.0 (10–10)	10.0 (10–10)	0.00	0.00	0.60	0.55
	Similarities	8.0 (7–9)	10.0 (9.25–10)	10.0 (9.5–10.5)	10.0 (9.5–11)	0.77 *	0.22	0.59 *	0.28
	Judgment	9.0 (9–10.5)	9.5 (9–10)	12.0 (10.5–12)	12.0 (9.5–12)	0.00	0.38	0.42	0.32

a = the scores represent average values and numbers in parentheses are ranges; b = the scores represent median values and numbers in parentheses are 25–75% quantile points; x, y, z = the effect size that resulted from attached t-test, Wilcoxon signed rank test, and Mann–Whitney’s *U* test, respectively; and * = *p* < 0.05.

**Table 3:** Comparison among indices associated with sleep and the number of sensor firings between BLp and RSp in two groups.

	≤23 Group			≥24 Group		
	BLp	RSp	Effect Size	BLp	RSp	Effect Size
Wake-up time	6:05 (0:19)	6:08 (0:19)	0.13	6:13 (0:33)	5:57 (0:34)	0.77 *
Bedtime	22:03 (0:25)	21:53 (0:18)	0.19	21:21 (0:47)	21:26 (0:45)	0.32
Sleep duration	7:52 (0:29)	7:59 (0:22)	0.06	9:24 (0:59)	8:38 (0:41)	0.89 *

The effect size with an asterisk indicates that the *p*-value was less than 5 %. Numbers in parentheses indicate the standard errors.

## Data Availability

The data presented in this study are available from the corresponding author on reasonable request.

## Supplementary Materials

The following are available online at https://www.mdpi.com/1660-4601/18/5/2498/s1, Table S1: Definitions of the other indices involving outings and toilet use, Table S2: Comparison of the other indices between BLp and RSp in the two groups.
