# Supplementary figures and images for: Effect of the Information Support Robot on the Daily Activity of Older People Living Alone in Actual Living Environment
Source: Int J Environ Res Public Health. 2021 Mar 3;18(5):2498. doi: 10.3390/ijerph18052498 (PMC7967636; doi:10.3390/ijerph18052498)

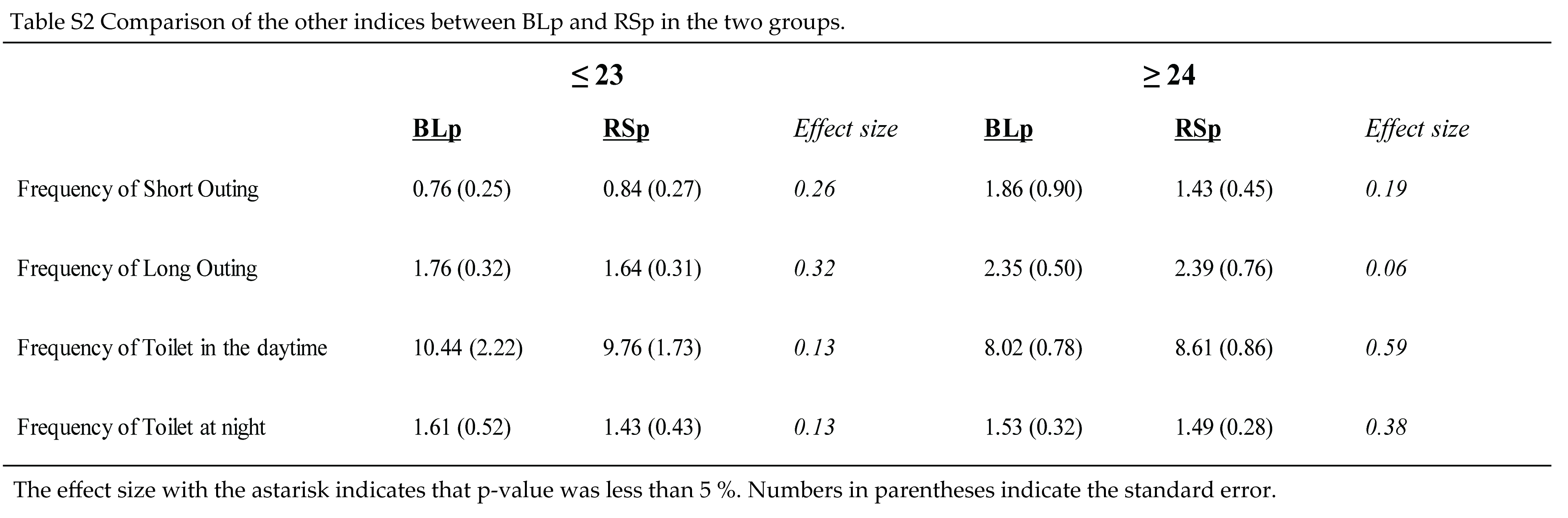

Supplement: Supplementary file 1 [file ijerph-18-02498-s001.zip › TableS2_IJERPH.tif]

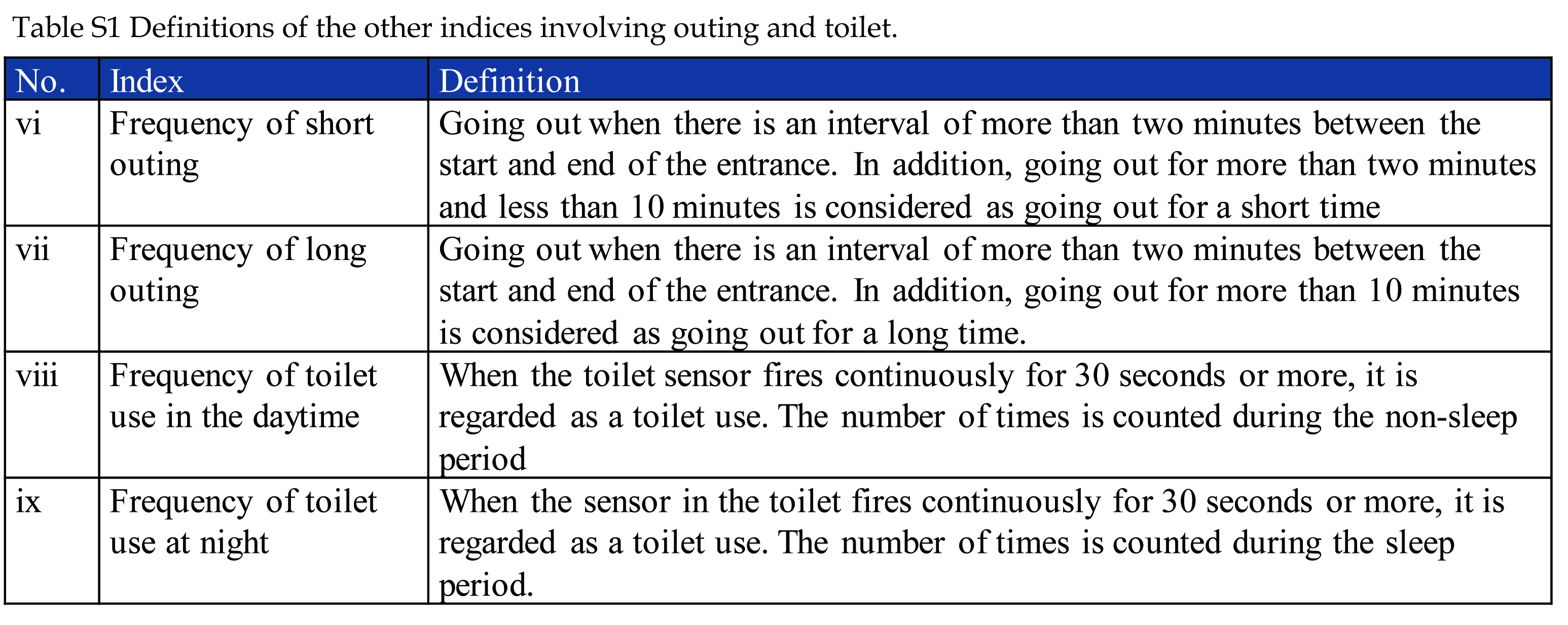

Supplement: Supplementary file 1 [file ijerph-18-02498-s001.zip › TableS1_IJERPH.tif]
